# Lewis Acid-Facilitated Radiofluorination of MN3PU: A LRRK2 Radiotracer

**DOI:** 10.3390/molecules25204710

**Published:** 2020-10-14

**Authors:** Noeen Malik, Shreya Bendre, Ralf Schirrmacher, Paul Schaffer

**Affiliations:** 1 Life Sciences Division, TRIUMF, 4004 Wesbrook Mall, Vancouver, BC V6T 2A3, Canada; noeen.malik@gmx.us (N.M.); schirrma@ualberta.ca (R.S.); 2 Molecular Oncology, BCCRC, 675 W 10th Ave, Vancouver, BC V5Z 1L3, Canada; sbendre@bccrc.ca; 3 Cross Cancer Institute, MICF, University of Alberta, 11560 University Ave, Edmonton, AB T6G 1Z2, Canada; 4 Department of Radiology, University of British Columbia, 2211 Wesbrook Mall, Vancouver, BC V6T 2B5, Canada; 5 Department of Chemistry, Simon Fraser University, 8888 University Drive, Burnaby, BC V5A 1S6, Canada

**Keywords:** Lewis acids, radiofluorination, nucleophilic aromatic substitutions, LRRK2

## Abstract

Background: Temperature-sensitive radiopharmaceutical precursors require lower reaction temperatures (<100 °C) during nucleophilic radiofluorination in order to avoid compound thermolysis, often resulting in sub-optimal radiochemical yields (RCYs). To facilitate nucleophilic aromatic substitution (S_N_Ar) of nucleofuges commonly used in radiofluorination (e.g., nitro group), we explored the use of Lewis acids as nucleophilic activators to accelerate [^18^F]fluoride incorporation at lower temperatures, and thereby increasing RCYs for thermolabile activated precursors. Lewis acid-assisted radiofluorination was exemplified on the temperature-sensitive compound 1-(4-(4-morpholino-7-neopentyl-7*H*-pyrrolo[2,3-d]pyrimidin-2-yl)phenyl)-3-(6-nitropyridin-3-yl)urea (MN3PU, compound 3) targeting leucine-rich repeat kinase 2 (LRRK2), an important target in the study of Parkinson’s disease and various cancers. Methods: To a vessel containing dried K[^18^F]F-K222 complex, a solution of precursor MN3PU ((3), 1 mg; 1.8 μmol) and Lewis acid (6 μL of 0.2 μmol: chromium II chloride (A), ferric nitrite (B) or titanocene dichloride (C)) in 500 μL of *N,N*-dimethylformamide (DMF) (with 10% *t*-BuOH for B) were added. Reactions were stirred for 25 min at 90 °C. In parallel, reactions were conducted without the addition of Lewis acids for baseline comparison. After purification via preconditioned Sep-Pak C18 plus cartridges, aliquots were analyzed by analytical radio-HPLC. Results: Non-decay corrected radiochemical yields (ndc RCYs) for [^18^F]FMN3PU (7) were improved from 1.7 ± 0.7% (no addition of Lewis acids) to 41 ± 1% using Cr(II) and 37 ± 0.7% using Ti(II)-based Lewis acids, with radiochemical purities of ≥96% and molar activities (A_m_) of up to 3.23 ± 1.7 Ci/μmol (120 ± 1.7 GBq/μmol). Conclusion: RCYs of [^18^F]FMN3PU (7) improved from ~5% using conventional nucleophilic radiofluorination, up to 41 ± 1% using Lewis-acid supported S_N_Ar.

## 1. Introduction

The production of [^18^F]fluorinated radiopharmaceuticals for positron emission tomography (PET) imaging typically involves syntheses via nucleophilic substitution (S_N_) [1]. The key requirements for nucleophilic aromatic substitutions (S_N_Ar) comprise a suitable leaving group (LG, e.g., NO_2_), a strong electron-withdrawing group (EWG) and a nucleophile (Nu) with either -*ortho* (2-position) or -*para* (4-position) orientation on the aromatic ring. Over the past few years, there has been tremendous development in radiofluorination chemistry involving both activated- and deactivated aryl-rings, including the deoxifluorination of phenols via Ru π-complexes [2], the substitution of aryl sulfonium salts [3], as well as iodonium, boronates or stannyl salts mediated by Cu, Ni or Pd catalysts [4].

In heteroaromatic compounds containing a pyridine ring, the N-atom exerts a considerable activating effect on the LG. Even in the presence of strong electron donating groups (EDG), such as -OCH_3_ and -CH_3_ at the -*para* or -*ortho* position to the LG, very high radiochemical yields (RCYs) (>80%) were observed at high temperatures (>110 °C) [5,6,7].

**Scheme 1:**
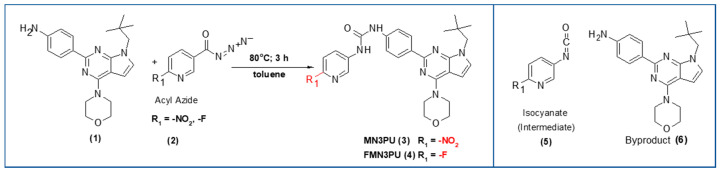
Syntheses of precursor and standard: Substituted aniline (**1**) (1.1 eq), toluene, azide (**3**, **4**) (1 eq), 80 °C, 3 h, inert atmosphere, yield >80% (*formation of urea bond* via *isocyanate*) (Red (R_1_): -F, -LG (-NO_2_)); byproduct (**6**) after thermal decomposition of precursor (MN3PU, 3) in solvents (DMF: *N,N*-dimethylformamide, DMSO: dimethyl sulfoxide).

Conventional S_N_Ar radiofluorination reactions are typically performed under conditions that enhance nucleophilic behavior of fluorine by removing water while providing a phase-transfer catalyst (PTC) to enhance fluoride activity in aprotic media. One of the most common examples includes the use of cryptand-potassium [^18^F]fluoride complex using 4,7,13,16,21,24- hexaoxa- 1,10- diazabicyclo [8.8.8] hexacosane (Kryptofix^®^) (K[^18^F]F-K222). In the current study, we sought to produce 1-(6-[^18^F]fluoropyridin-3-yl)-3-(4-(4-morpholino-7-neopentyl-7*H*-pyrrolo[2,3-d]pyrimidin-2-yl)phenyl)urea ([^18^F]FMN3PU, **7**) from 1-(4-(4-morpholino-7-neopentyl-7*H*-pyrrolo[2,3-d]pyrimidin-2-yl)phenyl)-3-(6-nitropyridin-3-yl)urea (MN3PU, 3) (Scheme 1). [^18^F]FM3PU (7) was designed to target leucine-rich repeat kinase 2 (LRRK2), based on kinase inhibitors as reported elsewhere [8]. The precursor possesses a -NO_2_ group as a LG -*ortho* (2 position) to the N-atom of the pyridine ring. However, our initial attempts utilizing conventional radiofluorination (90 °C for 25 min) were met with low RCYs (1.7 ± 0.7%), likely the result of pronounced thermolysis. In order to improve RCYs, we hypothesized that the use of Lewis acids such as chromium (II) chloride (A), ferric nitrite (B), and titanocene dichloride (C), may facilitate [^18^F]fluorination at lower reaction temperatures (<100 °C).

## 2. Results

Organic syntheses of both the precursor (MN3PU, 3) and the non-radioactive standard (FMN3PU, 4) were performed as reported elsewhere [4]. Briefly, the products were formed after the reaction of substituted aniline (1) with isocyanate intermediates ((5), formed in situ by heating the azide (2)) (Scheme 1). The reaction (i) provided both compounds 3 and 4 in yields of >80%.

**Figure 1:**
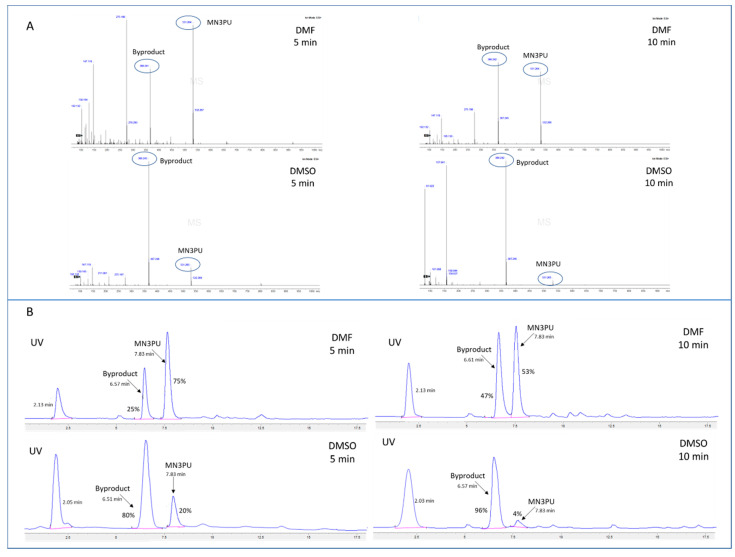
(**A**) LC/MS (+ESI) mass spectra analysis of aliquots from a solution containing MN3PU (compound **3**) in 500 µL of DMF after heating at 110 °C for 5 or 10 min shows rapid thermolysis of precursor in DMSO compared to DMF (MN3PU (**3**) mass: 531.26 [M + H]^+^; Byproduct (**6**) mass: 366.24 [M + H]^+^); (**B**) HPLC chromatograms demonstrating byproduct formation from heated MN3PU (**3**) in DMF or DMSO after 5 and 10 min (eluent (51% ACN in water (0.1% TFA), Phenomenex Luna (250 × 4.6 mm), Flow rate (1 mL/min) for 20 min, UV (254 nm)); (UV peak at 2.3 min might be of 2-nitro-4-aminopyridine).

Initial experiments using conventional radiofluorination methods resulted in very poor RCY (1.1 ± 1% at 120 °C and 10 min). With the rapid evolution of a UV-visible byproduct (R_t_: 6.7 min in HPLC) (Figure 1), we further investigated the thermal stability of MN3PU (3). Our results show that the precursor MN3PU (3) underwent thermal decomposition in polar, aprotic solvents (*N,N*-dimethylformamide (DMF), dimethyl sulfoxide (DMSO)) at high reaction temperature (~110 °C) as evidenced by the formation of one byproduct (6, observed as a significant color change, and the advent of a new UV peak in the HPLC of the crude reaction mixture). Further characterization of this byproduct (6) via LC/MS confirmed thermolysis of the urea bond (Mass: 366.24 [M + H]^+^), with the byproduct comprising between 50 and 90% (percentage area of peak in HPLC) of the solution in DMSO and DMF, respectively, within 10 min of heating (Scheme 1, Figure 1).

**Figure 2:**
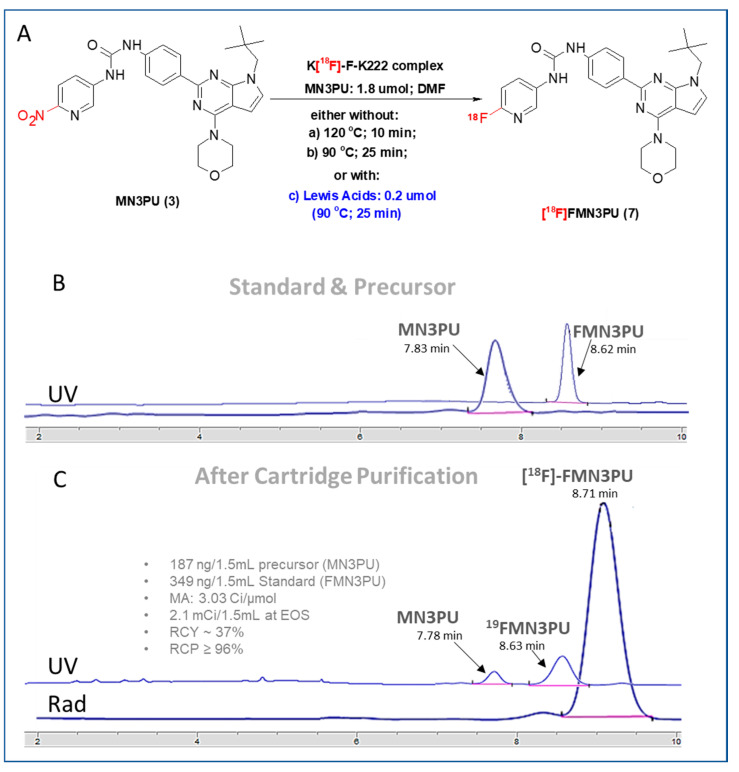
(**A**) Modified radiofluorination conditions of conventional methodology, either without (reaction conditions: (**a**) and (**b**)), or with (reaction conditions: (**c**)) Lewis acids titanocene dichloride, ferric nitrite, or chromium (II) chloride; (**B**) and (**C**) HPLC chromatograms (Standard and precursor (top B); QC (bottom, C); isocratic) for experiment with titanocene dichloride: Eluent (51% ACN in water (0.1% TFA), Phenomenex Luna (250 × 4.6 mm), Flow rate (1 mL/min) for 20 min, UV (254 nm) (Total amount of carrier and prcursor: 536 ng (non-radioactive ^19^FMN3PU (**4**) and MN3PU (**3**)).

In order to avoid thermolysis, we sought to perform the synthesis of [^18^F]FMN3PU (7) at a lower temperature (<100 °C) (Figure 2A (reaction conditions-b)), Table 1) under similar experimental conditions to those of the conventional radiofluorination method. However, the RCYs remained low (1.7 ± 0.7 at 25 min) and no byproduct was observed.

Moving forward, we attempted to improve RCYs by adding Lewis acids in reaction mixture. Significant improvements were observed upon introducing chromium (II) chloride (A) and titanocene dichloride (C), non-decay corrected (ndc) RCYs at 25 min were improved to 41 ± 1% and 37 ± 0.7%, respectively (Figure 2A). Reaction solutions containing chromium II chloride (A) or ferric nitrite (B) were opaque in DMF (but demonstrated better solubility in 10% *t*-BuOH in DMF), while solutions containing titanocene dichloride (C) remained clear due to the increased solubility of C in this solvent. In contrast, ndc RCYs with ferric nitrite (B) showed only marginal improvement (10 ± 1.2%) (Figure 2A, Table 1).

We observed a total of 341 ± 1.1 ng of FMN3PU in the final product sample (n = 4) giving a molar activity of 3.23 ± 1.7 Ci/µmol (120 ± 1.7 GBq/µmol; n = 4) at EOS. Total radiopharmaceutical preparation time (overall), including synthesis and formulation was 55 min.

## 3. Discussion

Decomposition of MN3PU (3) via thermolytic degradation of the urea moiety in polar-aprotic solvents (DMF/DMSO) and at higher temperatures (>100 °C), led to significant challenges in obtaining adequate RCYs for subsequent biodistribution studies. We were not able to obtain higher RCYs as reported for similar small-molecule tracers reported elsewhere [5,6,7].

**Scheme 2:**
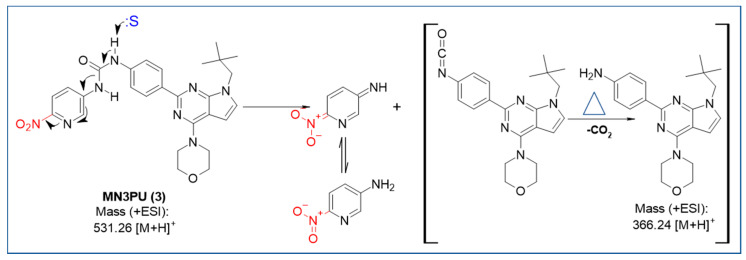
Proposed solvent (:S) participation in thermolysis of MN3PU (**3**) at high temperature (110 °C). NMR was used to identify byproduct (**6**) after separating using HPLC (See NMR in Supplementary Information).

For MN3PU (3), compound stability is likely challenged by the presence of a thermally sensitive urea group on the aromatic ring. This observed thermolysis can be attributed to the catalytic effect of polar aprotic solvents such as DMF and DMSO [9]. The catalytic behavior of such solvents can be attributed to specific characteristics such as dipole moment and steric hindrance [9]. In the current study, we hypothesize that solvent-mediated catalysis may be the reason for accelerated byproduct (6) formation via isocyanate intermediate at higher temperatures (110 °C). The proposed mechanism of this possible side reaction is shown in Scheme 2.

Thus, in order to improve yields at lower temperatures, i.e., <100 °C, we sought a better strategy, and we hypothesized that the addition of a Lewis acids could improve yields further. Our rationale behind using Lewis acids was to create ionic electrostatic interactions with -NH moiety of urea present at -*para* position to the LG which may enhance the nucleophilicity of LG by exerting a strong auxiliary effect, and may mediate the substitution of -NO_2_ group (LG) by [^18^F]fluoride (Nu). This hypothesis was proven correct when improved RCYs were observed after radiofluorinations with Lewis acids such as chromium (II) chloride (A) and titanocene dichloride (C). Some improvement to RCYs were observed when using ferric nitrite (B, 10 ± 1.2% compared to 1.7 ± 0.7), however yields were not as high as with other Lewis acids (A and C) (Table 1).

## 4. Materials and Methods

### 4.1. General

All chemicals, reagents, and solvents (chemical purity ˃95%) were purchased from Sigma-Aldrich (Oakville, ON, Canada) and used as received. NMR spectra for all products were recorded on a Bruker AvanceTM (II 600 MHz, III 500, or 400 MHz, Bruker BioSpin, Milton, ON, Canada) (equipped with a QNP or TCI cryoprobe). Mass samples were measured by LC/MS (Agilent™ Time-of-Flight, (Model 6210, Agilent Technologies, Mississauga, ON, Canada) using a Halo-C18 analytical column (2.1 × 50 mm) and gradient elution by water-acetonitrile (5 mM NH_4_OAc) via direct infusion positive mode acquisition method (ESI). Purification of non-radioactive compounds was done by automated flash chromatography (CombiFlash Rf+ system). Analytical radio-HPLC was carried out on an Agilent 1200 (Agilent Technologies, Mississauga, ON, Canada) equipped with a diode array detector and Raytest GABI Star NaI scintillation detector. Spectral analyses were performed using ACD2019-Spectrus Processor (Advanced Chemistry Development, Inc., Toronto, ON, Canada).

### 4.2. Chemistry

#### 4.2.1. Urea Formation 

*General, Syntheses of Compounds* (**3**, **4**), 4-(4-morpholino-7-neopentyl-7*H*-pyrrolo[2,3-d]pyrimidin-2-yl)aniline (**1**) (1.25 g, 3.41 mmol; 1.1 eq) dissolved in toluene (3 mL/125 mg of 1) was added to the solution of azide (2, 3.07 mmol; 1 eq; -NO_2_: 592 mg, -F: 509 mg) in toluene (10 mL) under inert atmosphere, and the reaction was heated at 80 °C for 3 h. At reaction completion, precipitate formation was observed, and workup commenced by rotary evaporation of the solvent, followed by addition of silica (~25% *w*/*w*) directly to the concentrated reaction residue. Dichloromethane (DCM) was added to the reaction-silica mixture to produce a slurry that was evaporated again, and the resulting fine powder was loaded into an empty Teledyne ISCO solid load cartridge. Purification was accomplished by automated flash normal-phase-chromatography y using gradient elution with Hexanes (A)/EtOAc (B) (10% to 95% of B). Upon elution, the solvent was evaporated under high vacuum with a liquid nitrogen cold trap to give a yellow powder.

*1-(4-(4-morpholino-7-neopentyl-7H-pyrrolo[2,3-d]pyrimidin-2-yl)phenyl)-3-(6-nitropyridin-3-yl)urea (MN3PU)* (**3**), ^1^H-NMR (DMSO-*d*_6_, 400 MHz): δ 9.78 (1H, s, NH), 8.77 (1H, s, NH), 8.36 (2H, d, *J* = 8.1 Hz, Ph), 8.16 (1H, d, *J* = 7.8 Hz, Ph), 8.16 (2H, d, *J* = 8.2 Hz, Ph), 7.60 (3H, d, *J* = 8.2 Hz, Pyridine), 7.23 1H, d, *J* = 7.5 Hz, 5,7-diazaindole), 6.68 (1H, d, *J* = 7.5 Hz, 5,7-diazaindole), 4.08 (2H, s, CH_2_), 3.95 (4H, t, *J* = 7.1, CH_2_, Morpholine), 3.78 (4H, t, *J* = 7.1 Hz, CH_2_, Morpholine), 0.96 (9H, s, CH_3_); ^13^C-NMR (DMSO-*d*_6_, 100 MHz): δ 157 (C-2N), 155 (C=O), 153 (C-2N), 152.8 (C-NO_2_), 141 (C-NH, Pyridine), 139 (CH, Pyridine), 138 (C-NH, Ph), 133 (CH, Pyridine), 129 (2CH, Ph), 128 (CH, 5,7-diazaindole), 127 (CH, Ph), 118 (CH, Pyridine), 116 (CH, Ph), 101 (CH, 5,7-diazaindole), 67 (CH_2_, Morpholine), 55 (2CH_2_, Morpholine), 46 (CH_2_, Morpholine), 34 (C-(CH_3_)_3_), 28 (3CH_3_); HRMS (+ESI) (*m*/*z*): found 531.25 [M + H]^+^, calc. 530.5783.

*1-(6-fluoropyridin-3-yl)-3-(4-(4-morpholino-7-neopentyl-7H-pyrrolo[2,3-d]pyrimidin-2-yl)phenyl)urea (FMN3PU)* (**4**), ^1^H-NMR (DMSO-*d*_6_, 400 MHz): δ 9.08 (1H, s, NH), 9.01 (1H, s, NH), 8.93 (1H, s, Pyridine), 8.35 (2H, d, *J* = 8.4 Hz, Ph), 8.28 (2H, d, *J* = 8.4 Hz, Ph), 8.08 (1H, m, *J* = 7.9, 2.1 Hz, Py), 7.56 (1H, d, *J* = 8.6 Hz, 5,7-diazaindole), 7.24 (1H, m, *J* = 7.7, 1.1 Hz, Pyridine), 6.67 (1H, d, *J* = 8.6 Hz, 5,7-diazaindole), 4.08 (2H, s, CH_2_), 3.94 (4H, t, *J* = 7.1, CH_2_, Morpholine), 3.78 (4H, t, J = 7.1 Hz, CH_2_, Morpholine), 0.97 (9H, s, CH_3_); ^13^C-NMR (DMSO-*d*_6_, 100 MHz): δ 157 (C-F), 153 (C-2N, Pyrimidine), 152 (C=O), 151 (C-NH, Ph), 142 (CH, Pyridine), 141 (CH, Pyridine), 138 (C-NH, Pyridine), 134 (C, Ph), 128 (CH, Ph), 127 (CH, 5,7-diazaindole), 120 (CH, Pyridine), 119 (CH, Ph), 101 (CH, 5,7-diazaindole), 67 (CH_2_, Morpholine), 55 (CH_2_), 46 (CH_2_, Morpholine), 34 (C), 28 (CH_3_); HRMS (+ESI) (*m*/*z*): found 504.24 [M + H]^+^, calc. 503.5712.

*4-(4-Morpholino-7-neopentyl-7H-pyrrolo[2,3-d]pyrimidin-2-yl)aniline (Thermal byproduct)* (**6**), ^1^H-NMR (DMSO-*d*_6_, 400 MHz): δ 8.33 (2H, d, *J* = 8.5 Hz, Ph), 8.28 (2H, d, *J* = 8.3 Hz, Ph), 6.94 (1H, d, *J* = 7.6 Hz, 5,7-diazaindole), 6.45 (1H, d, *J* = 7.5 Hz, 5,7-diazaindole), 5.58 (2H, s, NH_2_), 4.00 (6H, q, *J* = 6.5 Hz, CH_2_), 3.87 (4H, t, *J* = 7.1, CH_2_, Morpholine), 0.99 (9H, s, CH_3_); ^13^C-NMR (DMSO-*d*_6_, 100 MHz): δ 157 (C-NH_2_), 155.44 (C-2N, Pyrimidine), 155.20 (C-2N, Ph), 153 (C-2N, Pyrimidine), 133 (C, Ph), 128 (2CH, Ph), 127 (CH, 5,7-diazaindole), 119 (2CH, Ph), 101 (CH, 5,7-diazaindole), 67 (2CH_2_, Morpholine), 55 (CH_2_), 45 (2CH_2_, Morpholine), 34 (C), 28 (CH_3_); HRMS (+ESI) (*m*/*z*): found 366.23 [M + 1]^+^, calc. 365.4719.

#### 4.2.2. [^18^F]Fluoride Production

No-carrier-added (n.c.a.) [^18^F]fluoride was produced using the TR13 cyclotron at TRIUMF via the ^18^O (p, n)^18^F reaction by irradiating 1.20 mL of ˃95% enriched [^18^O] water with 13 MeV protons.

*[^18^F]FMN3PU* (**7**), [^18^F]Fluoride fixed on preconditioned QMA light cartridge (0.5 M K_2_CO_3_/Water) was eluted by 1.2 mL of K_2_CO_3_/K222 in MeCN (3.5 mg K_2_CO_3_/500 µL and 7 mg K222/700 µL) into reaction vial. After azeotropic drying at 110 °C for 15 min, a solution of precursor (MN3PU, 3; 1 mg; 1.8 µmol) and Lewis acid (6 µL of 0.2 µmol, chromium II chloride (A) or ferric nitrite (B) or titanocene dichloride (C)) in 500 µL of DMF (with 10% *t*-BuOH for A and B) was added to reaction vial and reaction mixture was stirred at 90 °C for 25 min (120 °C for 10 min and 90 °C for 25 min without Lewis acids). Afterwards, the reaction mixtures were diluted using 20 mL of distilled, deionized water into round bottom flask and passed through preconditioned C18 plus (Sep-Pak, Waters; EtOH/Water), washed with 10 mL water, and eluted using 1 mL of injection solution (0.3 mL PEG/0.1 mL EtOH/0.1 mL DMSO + 0.5 mL PBS). All RCYs in the present work were non-decay corrected (ndc) for a total synthesis time of 55 min. RCYs without Lewis acids were 1.1 ± 1% (120 °C for 10 min) and 1.7 ± 0.7% (90 °C for 25 min), whereas with Lewis acids, yields were 10 ± 1.2% when using ferric nitrie (B), 41 ± 1% when using chromium II chloride (A) and 37 ± 0.7% when using titanocene dichloride (C). Radiochemical purity (RCP) was ≥ 96% and molar activity was 3.23 ± 1.7 Ci/μmol (120 ± 1.7 GBq/μmol) (n = 4). In addition to the carrier (341 ± 1.1 ng/1.5 mL), the only significant impurity in the final tracer preparations was the radiolabeling precursor (MN3PU, compound 3, 180 ± 0.9 ng).

## 5. Conclusions

In the present study, it was observed that the addition of certain Lewis acids led to significantly improved RCYs for [^18^F]FMN3PU (7) when compared to yields obtained using conventional nucleophilic radiofluorination methods. These results suggest that the addition of Lewis acids may provide improved radiofluorination yields for compounds with structural similarities to that of MN3PU (3).

## Figures and Tables

**Table 1:** Comparison of radiochemical yields (RCYs) via different methodologies (Figure 2A)

Experiments (n = 4)	Conventional (Reaction Conditions-b)	Lewis Acids (Reaction Conditions-c)		
		Chromium II Chloride (A)	Ferric Nitrite (B)	Titanocene Dichloride (C)
90 °C (25 min)	1.7 ± 0.7%	41 ± 1%	10 ± 1.2%	37 ± 0.7%

## Supplementary Materials

The following are available online. Spectral analyses (NMR and mass spectra) of MN3PU (3), FMN3PU (4), and byproduct (6) can be found in the supplementary information.
