# Supplementary material for: Lewis Acid-Facilitated Radiofluorination of MN3PU: A LRRK2 Radiotracer
Source: Molecules. 2020 Oct 14;25(20):4710. doi: 10.3390/molecules25204710 (PMC7587332; doi:10.3390/molecules25204710)
Supplement: Supplementary file 1 [file molecules-25-04710-s001.pdf]

Supplementary Data

Lewis Acid-facilitated Radiofluorination of MN3PU: A LRRK2 Radiotracer

Noeen Malik [1], Shreya Bendre[2], Ralf Schirrmacher [1,3] and Paul Schaffer [1,4,5] *

1 Life Sciences Division, TRIUMF, noeen.malik@gmx.us (N.M.); pschaffer@triumf.ca (P.S.)

2 Molecular Oncology, BCCRC, sbendre@bccrc.ca (S.B.)

3 Cross Cancer Institute, MICF, University of Alberta, schirrma@ualberta.ca (R.S.)

4 Department of Radiology, University of British Columbia

5 Department of Chemistry, Simon Fraser University

* Correspondence: pschaffer@triumf.ca (P.S.); Tel.: +1-604-222-7696

NMR and Mass Spectra

MN3PU (3), FMN3PU (4), Thermal byproduct (6)

(ACD2019-Spectrus Processor)

MN3PU (3)

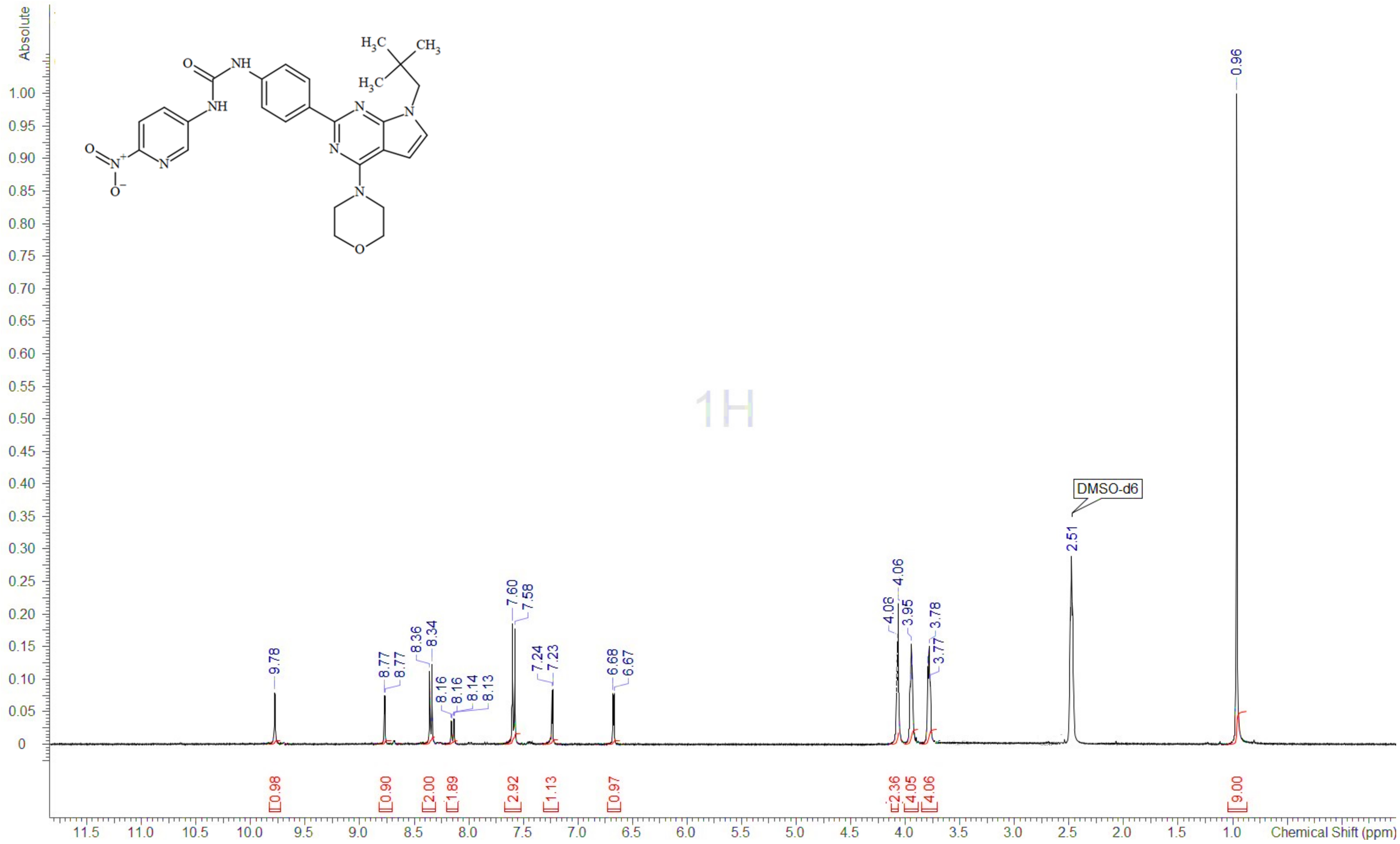

Absolute
1H
DMSO-d6
9.78
8.77
8.77
8.36
8.34
8.16
8.16
8.14
8.13
7.60
7.58
7.24
7.23
6.68
6.67
4.06
4.06
3.95
3.78
3.77
2.51
0.96
0.98
0.90
2.00
1.89
2.92
1.13
0.97
2.36
4.05
4.06
9.00
Chemical Shift (ppm)

MN3PU (3)

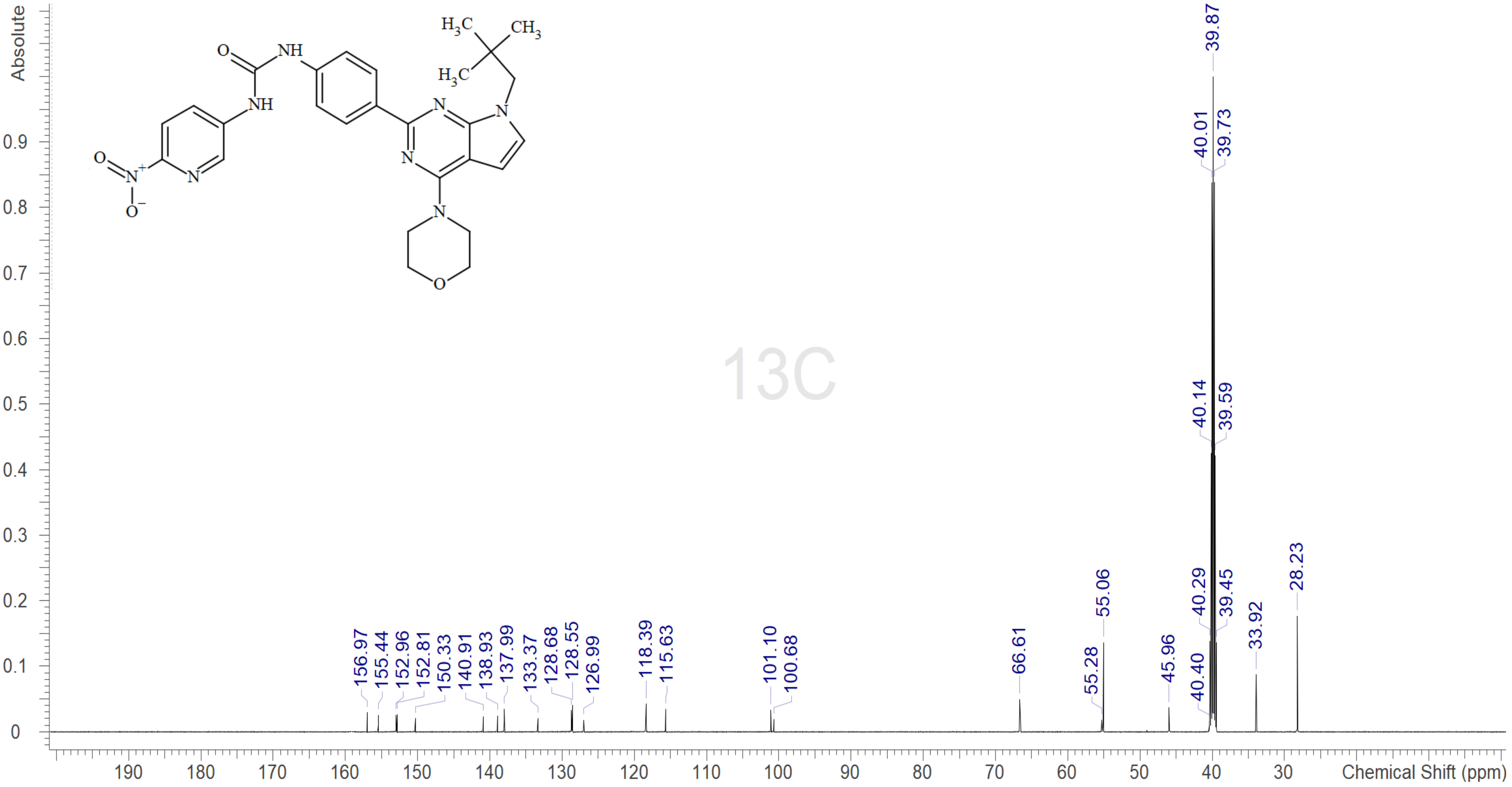
Absolute
13C
156.97
155.44
152.96
152.81
150.33
140.91
138.93
137.99
133.37
128.68
128.55
126.99
118.39
115.63
101.10
100.68
66.61
55.28
55.06
45.96
40.40
40.29
40.14
40.01
39.87
39.73
39.59
39.45
33.92
28.23
Chemical Shift (ppm)

MN3PU (3) (LC/MS)

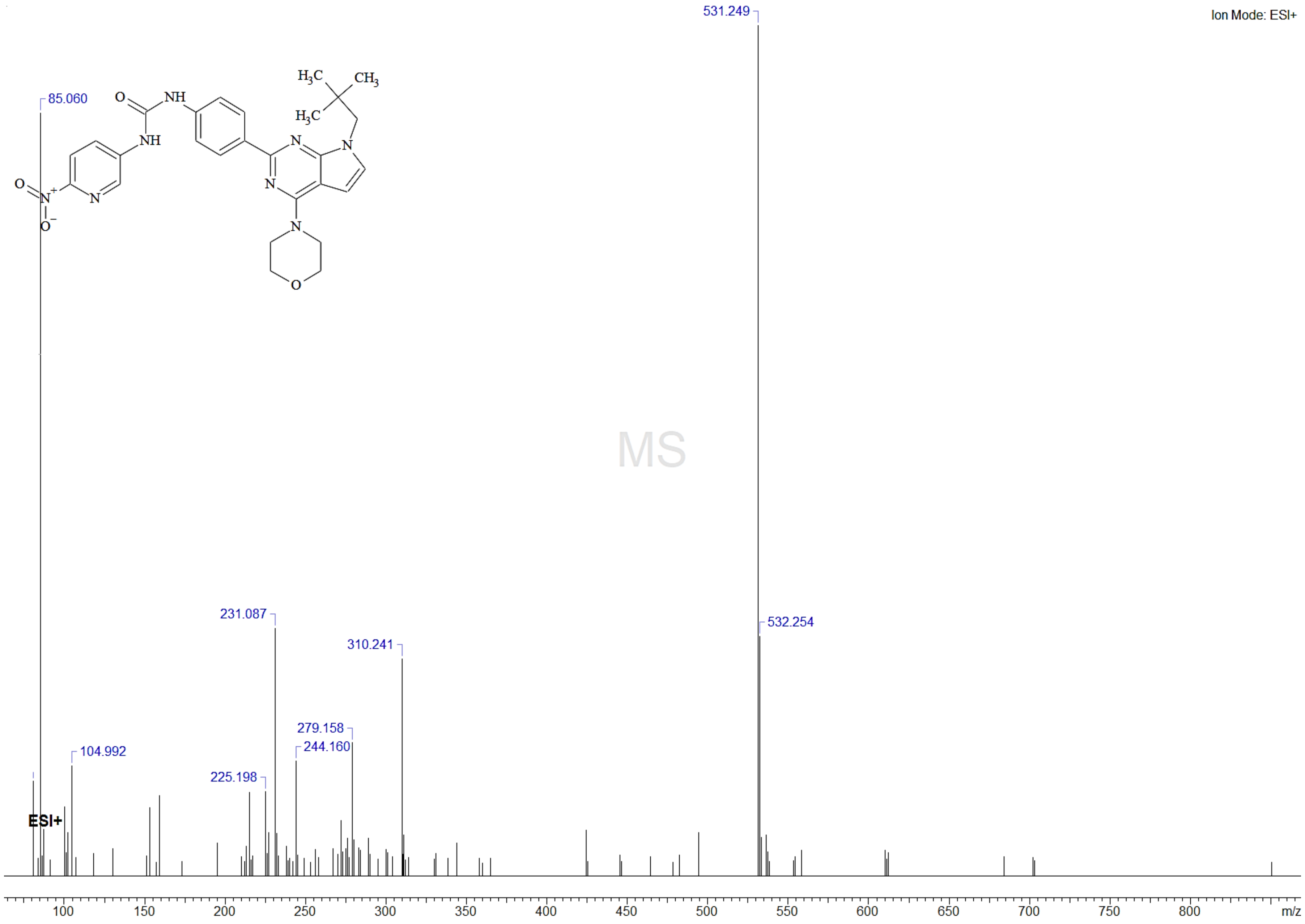

Ion Mode: ESI+
531.249
85.060
532.254
231.087
310.241
279.158
244.160
104.992
225.198
ESI+
MS
100
150
200
250
300
350
400
450
500
550
600
650
700
750
800
m/z

MN3PU (3) (HRMS)

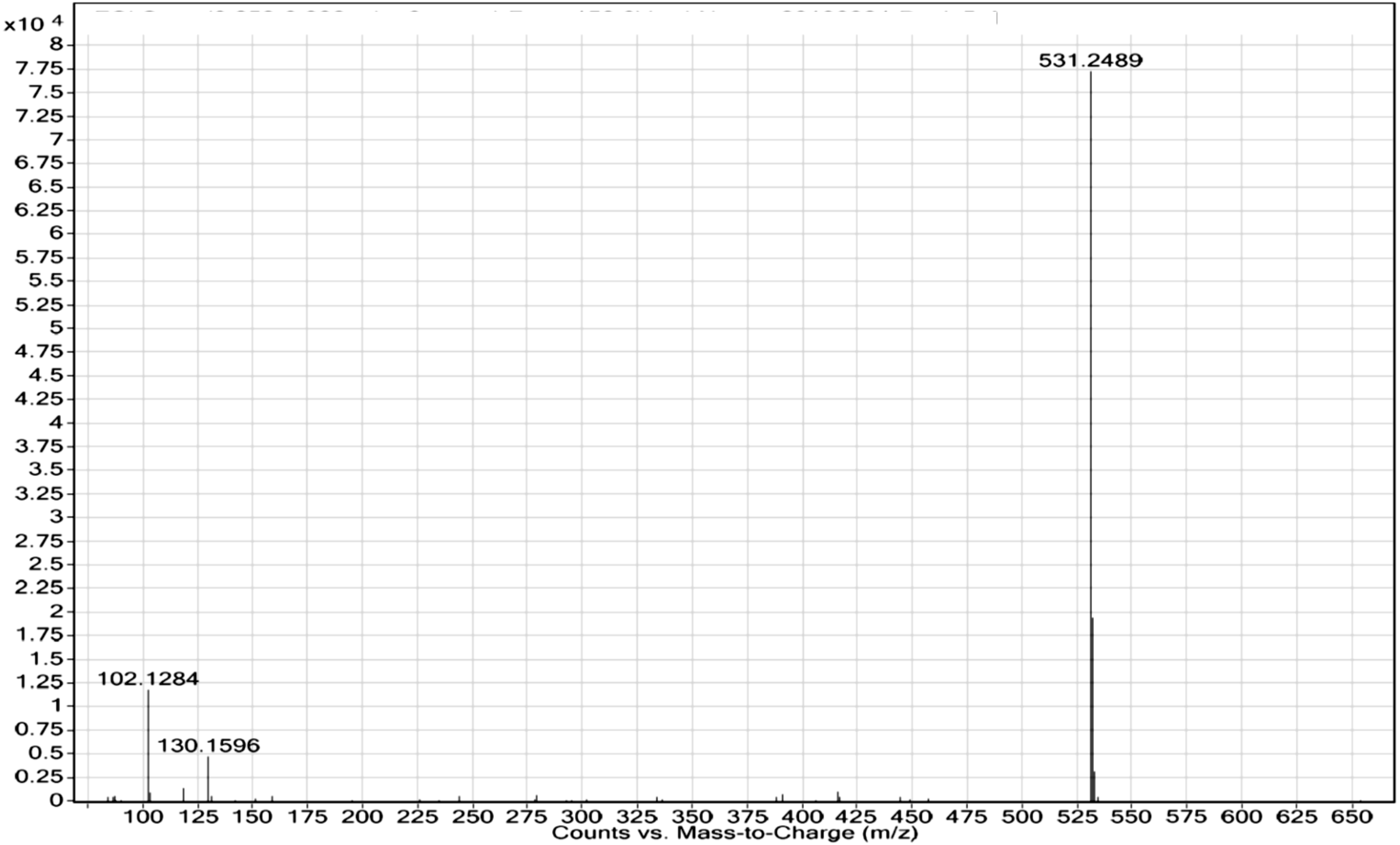
x10 4
8
7.75
7.5
7.25
7
6.75
6.5
6.25
6
5.75
5.5
5.25
5
4.75
4.5
4.25
4
3.75
3.5
3.25
3
2.75
2.5
2.25
2
1.75
1.5
1.25
1
0.75
0.5
0.25
0
531.2489
102.1284
130.1596
100 125 150 175 200 225 250 275 300 325 350 375 400 425 450 475 500 525 550 575 600 625 650
Counts vs. Mass-to-Charge (m/z)

FMN3PU (4)

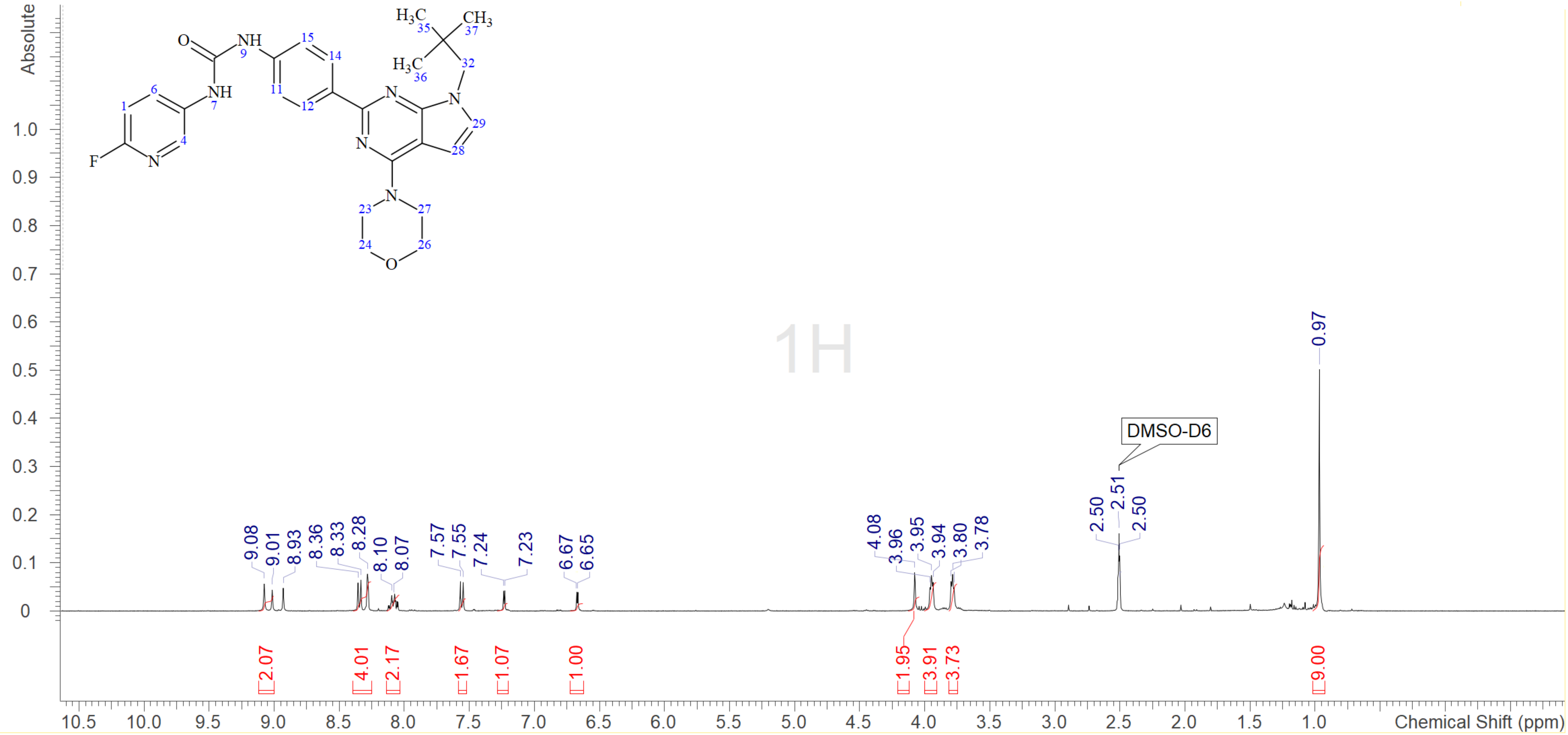

Absolute
1H
DMSO-D6
9.08 9.01 8.93 8.36 8.33 8.28 8.10 8.07 7.57 7.55 7.24 7.23 6.67 6.65
4.08 3.96 3.95 3.94 3.80 3.78 2.50 2.51 2.50 0.97
2.07 4.01 2.17 1.67 1.07 1.00 1.95 3.91 3.73 9.00
Chemical Shift (ppm)

FMN3PU (4)

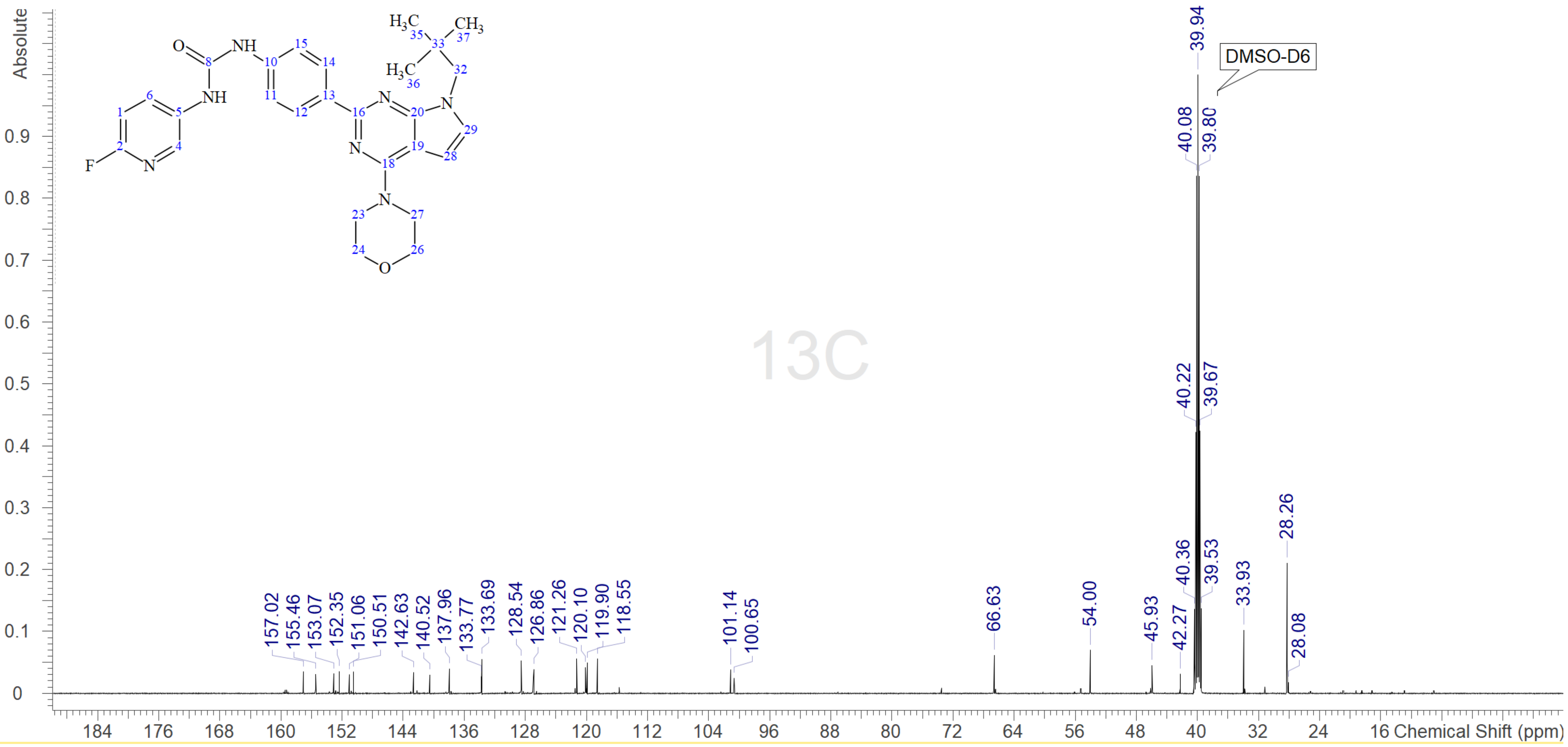

13C
DMSO-D6
Absolute
Chemical Shift (ppm)
157.02
155.46
153.07
152.35
151.06
150.51
142.63
140.52
137.96
133.77
133.69
128.54
126.86
121.26
120.10
119.90
118.55
101.14
100.65
66.63
54.00
45.93
42.27
40.36
40.22
40.08
39.94
39.80
39.67
39.53
33.93
28.26
28.08

FMN3PU (4) (LC/MS)

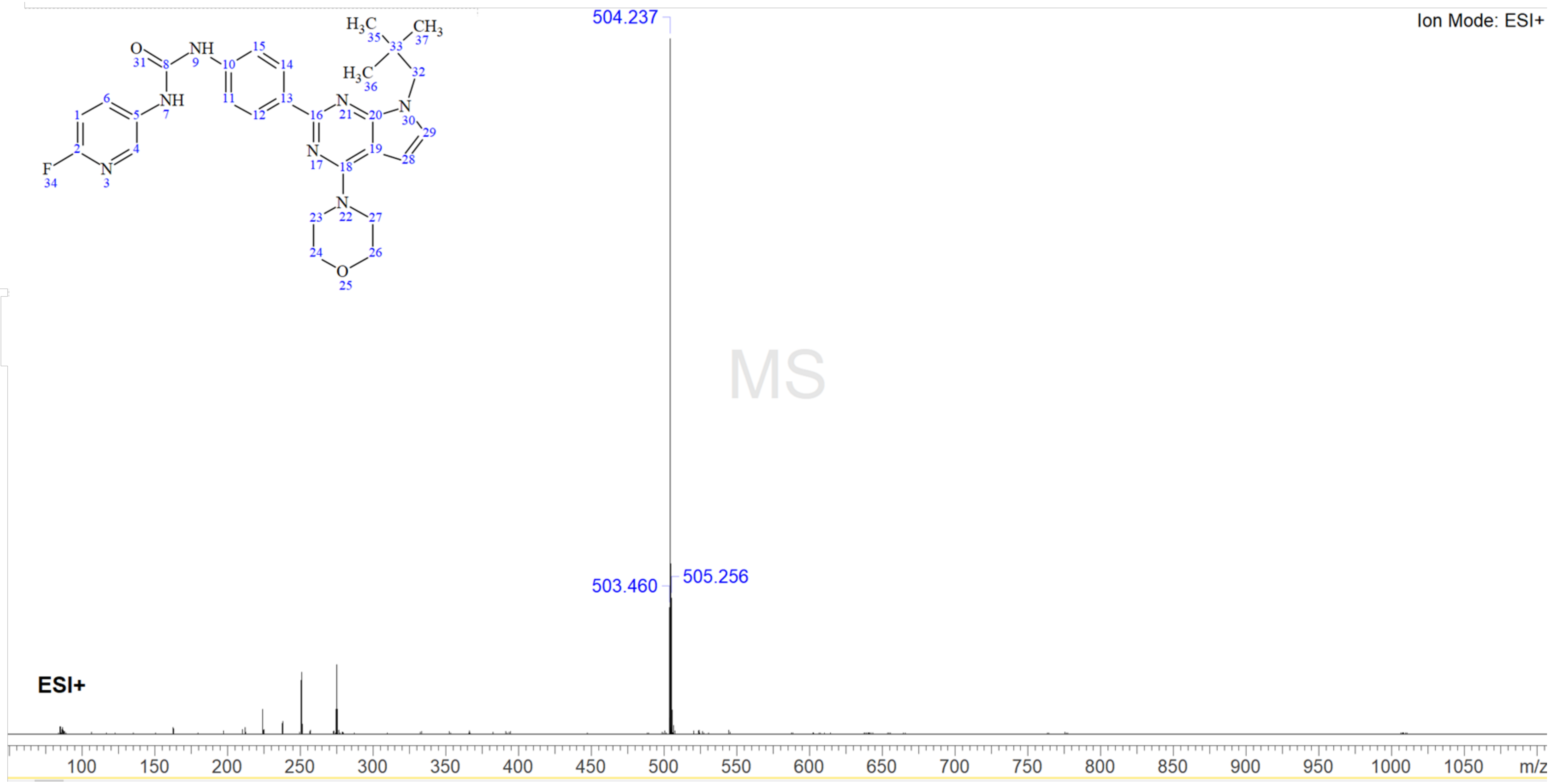

FMN3PU (4) (HRMS)

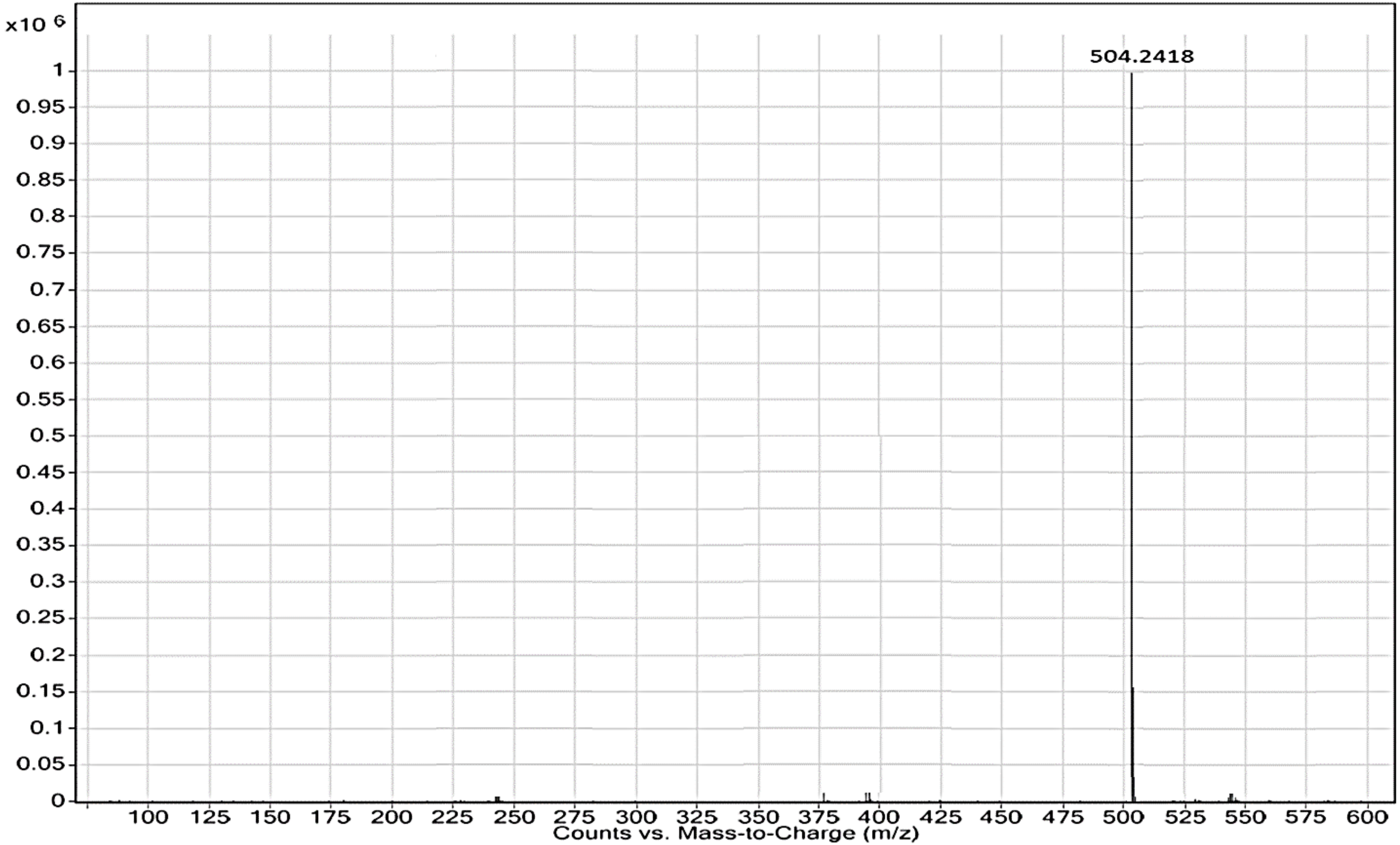

Thermal Byproduct (6)

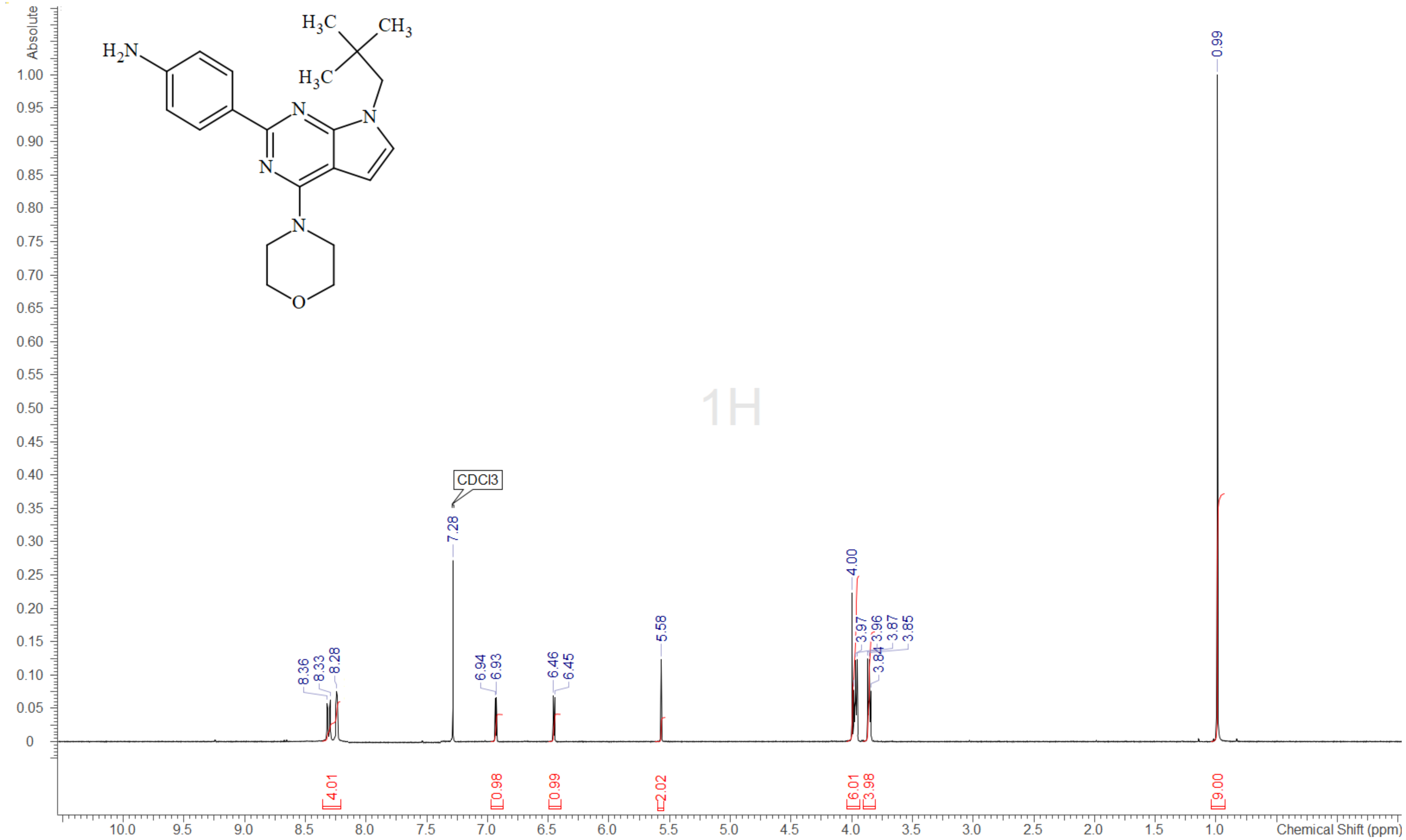

Thermal Byproduct (6)

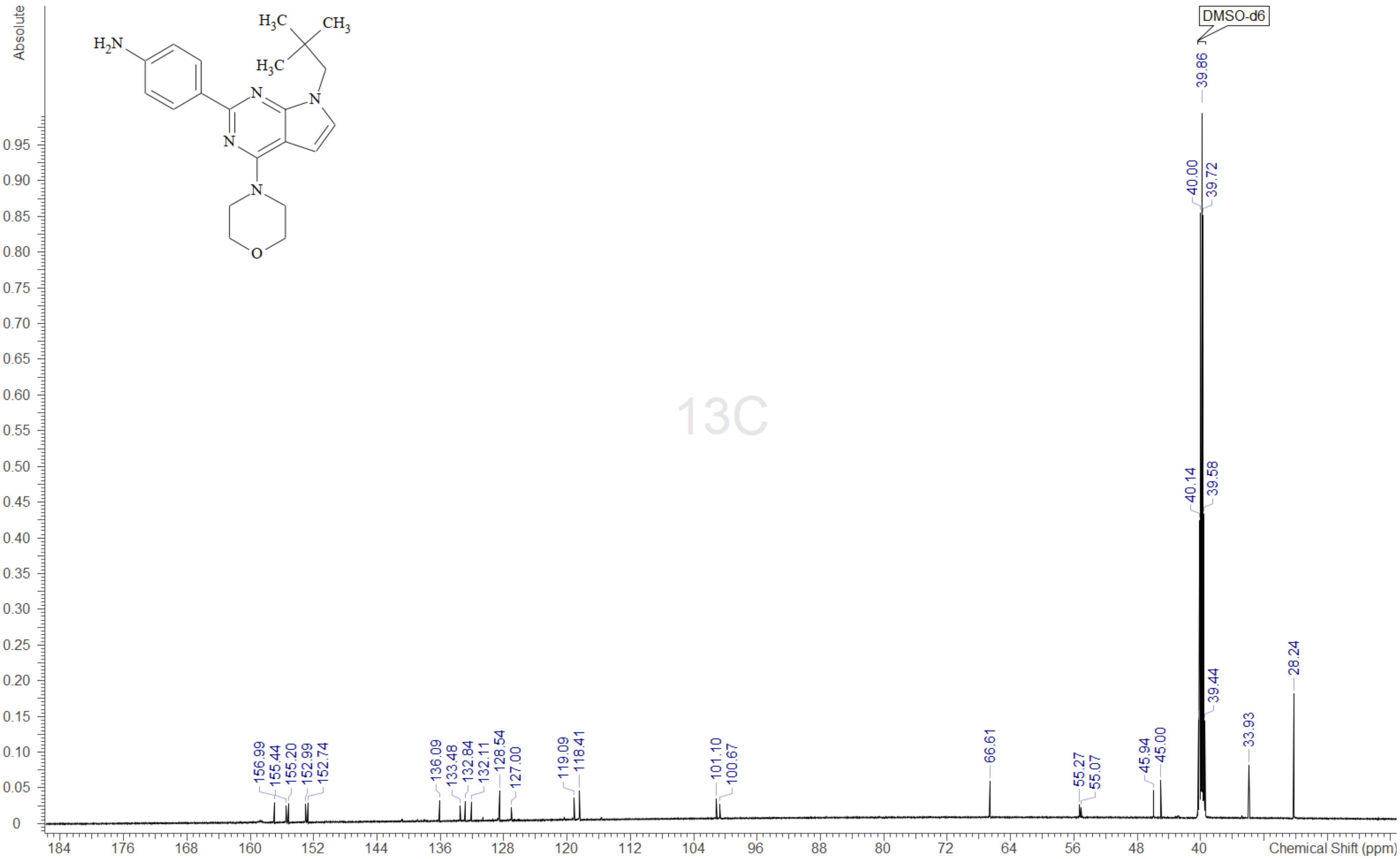

Thermal Byproduct (6) (LC/MS)

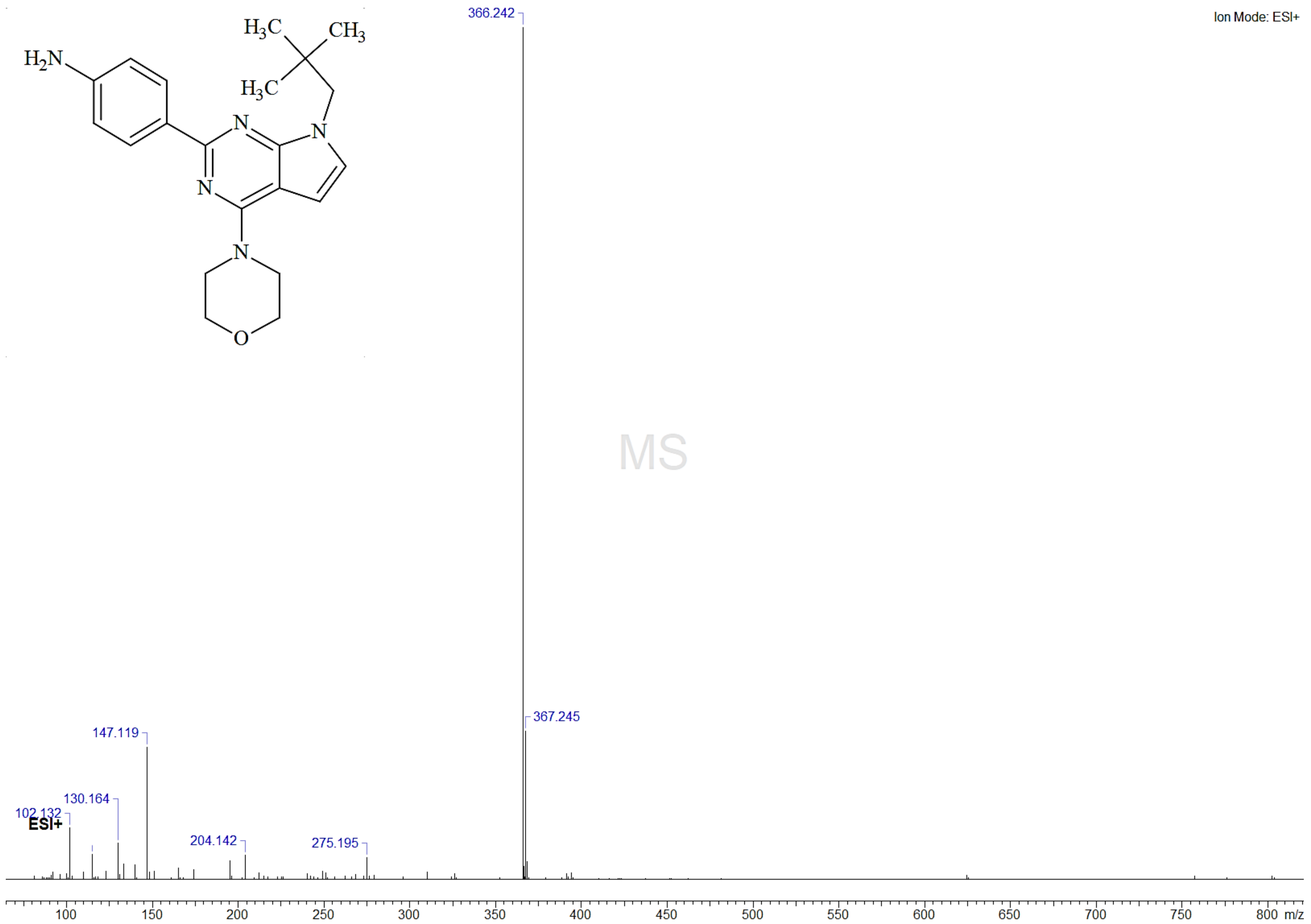

Thermal Byproduct (6) (HRMS)

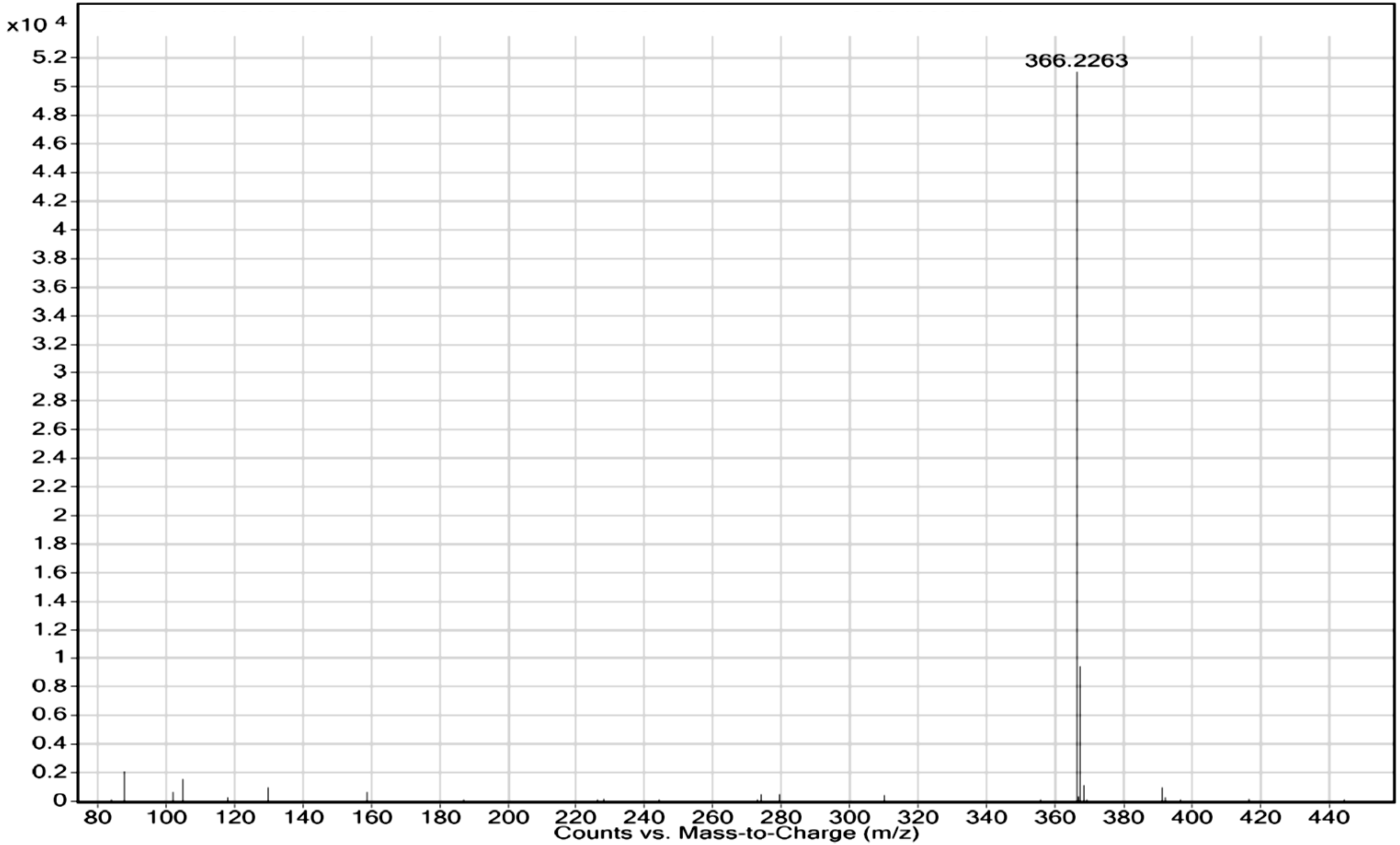

Calibration Curves

MN3PU (3), FMN3PU (4)

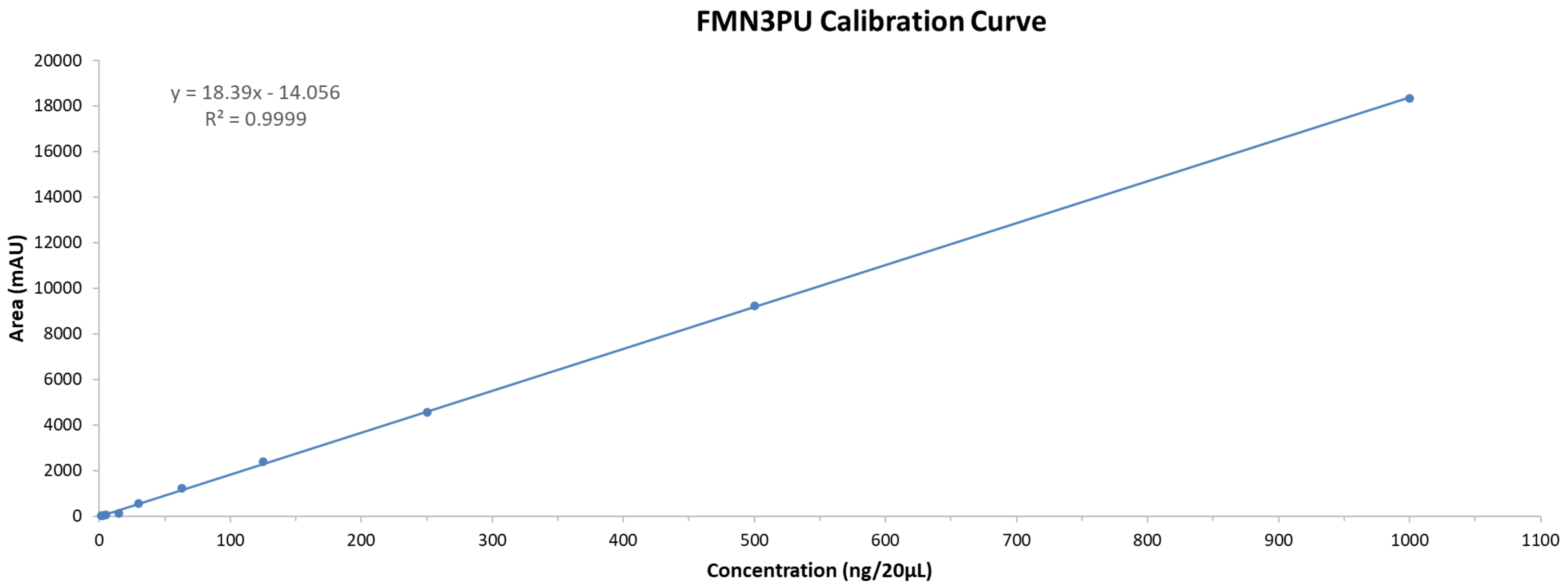
FMN3PU Calibration Curve
y = 18.39x - 14.056
R² = 0.9999
Area (mAU)
20000
18000
16000
14000
12000
10000
8000
6000
4000
2000
0
0
100
200
300
400
500
600
700
800
900
1000
1100
Concentration (ng/20µL)

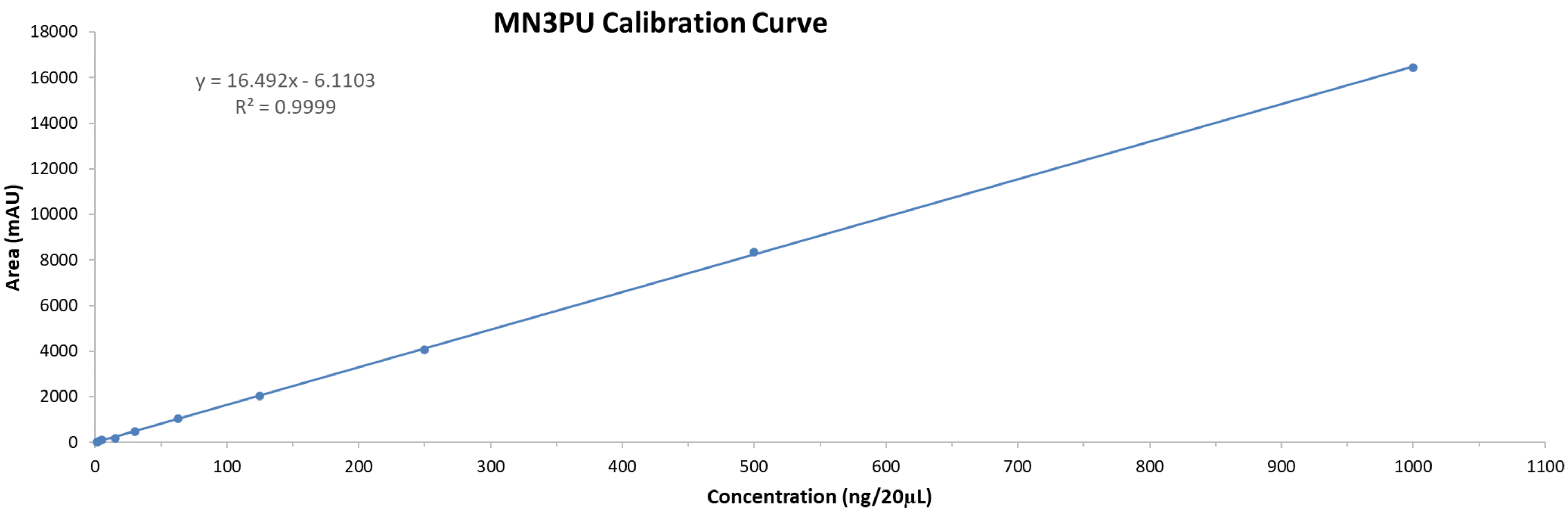
MN3PU Calibration Curve
y = 16.492x - 6.1103
R² = 0.9999
Area (mAU)
Concentration (ng/20μL)
18000
16000
14000
12000
10000
8000
6000
4000
2000
0
0
100
200
300
400
500
600
700
800
900
1000
1100
